# Creating equitable healthcare quality and safety for children with intellectual disability in hospital

**DOI:** 10.1111/cch.12787

**Published:** 2020-06-05

**Authors:** Laurel Mimmo, Susan Woolfenden, Joanne Travaglia, Reema Harrison

**Affiliations:** ^1^ Health Management, School of Public Health and Community Medicine, Faculty of Medicine University of New South Wales Sydney New South Wales Australia; ^2^ Clinical Governance Unit Sydney Children's Hospitals Network Sydney New South Wales Australia; ^3^ School of Women’s and Children’s Health, Faculty of Medicine University of New South Wales Sydney New South Wales Australia; ^4^ Community Child Health Sydney Children's Hospital Randwick New South Wales Australia; ^5^ Health Services Management, Centre for Health Services Management, Faculty of Health University of Technology Sydney Sydney New South Wales Australia

**Keywords:** health services research, intellectual disability, paediatrics, partnership

## Abstract

Children with intellectual disability are susceptible to poor experiences of care and treatment outcomes, and this may compound existing health inequities. Evidence to date indicates three priority areas that must be addressed in order to reduce these inequities in the safety and quality of care for children with intellectual disability. Firstly, we need reliable methods to identify children with intellectual disability so that healthcare organizations understand their needs. Secondly, we need to develop quality metrics that can assess care quality and unwarranted care variation for children with intellectual disability in hospital. Finally, for a comprehensive understanding of the safety and quality of care for these children, and how to improve, it is critical that healthcare organizations partner with parents/carers and enable children with intellectual disability to voice their experiences of care. Children with intellectual disability have higher healthcare utilization than their peers; yet, their voice is rarely sought to optimize the safety and quality of their healthcare experience. Patient experience narratives enhance our understanding of the genesis of adverse events. By addressing these priorities, children with intellectual disability will be identified, and health services will measure and understand the problematic and beneficial variations in care delivery and can then effectively partner with children and their parents/carers to address the inequities in care quality and create safer healthcare.

**Key Messages**

- Children with intellectual disability are susceptible to poorer quality of care than their peers when they are admitted to hospital, and this may compound existing health inequities for this group. To address this, we must identify these children when they access tertiary healthcare, meaningfully measure their quality of care and enable the patient voice to help optimize their healthcare experience.
- ICD‐10 codes are not reliable for identifying children with intellectual disability when they are admitted to hospital; reliable identification of children with intellectual disability is the first step in understanding where and how clinical variation occurs.
- Understanding of the patient and system factors underlying unwarranted clinical variation for children with intellectual disability can enable healthcare organizations to adapt care delivery to ensure high‐quality services to meet the needs of the child with intellectual disability.
- Partnering with these children and their carers to hear their experiences of care will enable healthcare organizations to call attention to and emulate examples of good care quality experiences for these children.

## 1 HEALTHCARE INEQUITIES AMONGST CHILDREN WITH INTELLECTUAL DISABILITY IN HOSPITAL

People with intellectual disability have higher rates of health care utilization (Bebbington, Glasson, Bourke, De Klerk, & Leonard, 2013; Heslop et al., 2014; Iacono, Bigby, Unsworth, Douglas, & Fitzpatrick, 2014), experience more preventable harms and poorer care quality than the general population (Heslop et al., 2014; Iacono et al., 2014) and may die up to 20 years earlier than their peers (Heslop et al., 2014; Trollor, Srasuebkul, Xu, & Howlett, 2017). In inpatient paediatric settings, quality and safety risks in children have been associated with medical complexity (Khan et al., 2016; Stockwell et al., 2018) and prolonged length of stay (Khan et al., 2016; Matlow et al., 2012). Children with intellectual disability have unique quality and safety risks in hospital, with particular vulnerability to communication and medication related errors (Meurer, Yang, Guse, Scanlon, & Layde, 2006; Taitz, 2015).

This presents a troubling conundrum: Children with intellectual disability who are most in need of specialist paediatric care also have a heightened risk of harm every time they access tertiary healthcare. Yet, research exploring the risks for this population is sparse. In addition, the cost of a hospital admission to children and their families from a social, educational and financial perspective compound the existing health and social inequities children with intellectual disability experience.

A comprehensive understanding of the causal links of the quality and safety risks, and the modifying effect on existing health inequities for children with intellectual disability, is lacking.

Our programme of research has highlighted three areas that must be addressed to provide the foundation for measuring, understanding and enhancing equity in the quality of care for children with intellectual disability. These are reliable identification; exploring indirect indicators of poor quality; and meaningful consumer engagement, reflecting the disability mantra ‘nothing about us without us’ (Iezzoni & Long‐Bellil, 2012) and Berwick's patient safety mantra ‘nothing about me without me’ (Berwick, 2009).

## 2 RELIABLE IDENTIFICATION OF CHILDREN WITH INTELLECTUAL DISABILITY

Differences in clinical outcomes that are problematic across patient populations are referred to as unwarranted clinical variation (Harrison et al., 2019). Identifying unwarranted clinical variation for children with intellectual disability requires baseline quality of care metrics and an awareness of the care delivery adaptions needed for the child, necessitating reliable methods to identify children with intellectual disability in medical records. Our recent study of admissions of inpatient children coded with intellectual disability to two tertiary children's hospital highlighted this; for the 336 children with intellectual disability identified, one third of their admissions did not have a code for intellectual disability (Mimmo, Woolfenden, Travaglia, & Harrison, 2019a). Others have reported similar challenges identifying children with intellectual disability using hospital morbidity data (Bourke, Wong, & Leonard, 2018). In the United Kingdom, Kenten et al. (2019) found inconsistent methods to identify children within medical records (Kenten et al.). The implications of flags or alerts for the quality and safety experience for children are not fully known; could it enhance care quality or homogenize this group, meaning the individual needs of each child are not considered? Furthermore, our study found documentation of intellectual disability is not standardized, and some routinely used abbreviations are dependent on the context. For example, the abbreviation ‘DD’ could mean developmental delay or developmental disability, differential diagnosis or dual diagnosis; ‘ASD’ can correctly indicate Autism Spectrum Disorder or a common cardiac condition of childhood, Atrial Septal Defect. Flags or alerts to identify intellectual disability rely on clinical staff specifying the clinical context to correctly interpret abbreviated terminology.

Clinical incident data systems do not capture patient characteristics such as intellectual disability; therefore, the unique risks for this patient cohort cannot be monitored. Furthermore, without an awareness of the individual factors that necessitate adjustments to care delivery for this patient group, we cannot distinguish between susceptibility to preventable harms and complications of treatment due to variations in care. For example, studies comparing post‐operative complications in children with and without down syndrome report higher rates of complications in children with down syndrome (Desai et al., 2014; Morabito, Lall, Gull, Mohee, & Bianchi, 2006; Travassos, van Herwaarden‐Lindeboom, & van der Zee, 2011), with one study noting that children with down syndrome ‘are predisposed to complications and thus warrant more cautious management’ (Morabito et al., 2006, p. 181).

How can, or do, healthcare organizations separate healthcare complications from preventable harm and/or care quality compromise? More importantly, without answers to these questions, we cannot distinguish unwarranted clinical variation from positive clinical variation, where standard care is adapted to enhance care quality and equity (Hollnagel, Wears, & Braithwaite, 2015). If healthcare organizations lack the capability to differentiate between variation that is problematic and that which is beneficent, good quality care may inadvertently be discouraged.

Healthcare staff may use covert workarounds to adapt care delivery believing this will provide safe and equitable care, particularly in the perioperative setting, with questionable benefit. For example, in a study of cleft palate surgery experiences for young people, healthcare staff reported using physical restraint with young people with intellectual disability and having a ‘lower threshold’ for giving general anaesthetic, for procedures that usually do not require sedation in their age‐equivalent peers (Bates, Forrester‐Jones & McCarthy, 2019, p288). Alternatively, Blair et al. identified a lack of reasonable adjustment to healthcare delivery as an opportunity to develop a procedure for safe adaptions to care for children with intellectual disability in the perioperative setting (Blair et al., 2017).

Thus, it is imperative that health systems accurately identify the inpatient paediatric intellectual disability population. This will allow for reliable systems to identify and measure the health utilization, care quality and patient safety outcomes for children with intellectual disability, creating health services which will meet their care needs.

## 3 EXPLORING INDIRECT INDICATORS OF POOR QUALITY CARE

Care quality metrics, such as length of stay and clinical outcomes, can provide an opportunity to identify and explore possible unwarranted clinical variation, through a comprehensive understanding of the underlying patient and system factors impacting on care delivery and outcomes (Harrison et al., 2019). Increased length of stay is an accepted indicator of healthcare acquired complications and quality (Organisation for Economic Cooperation and Development, 2015). Health services globally routinely use length of stay to benchmark variance in clinical outcomes for different patient cohorts and between health services (Organisation for Economic Cooperation and Development, 2015), including in the inpatient paediatric population.

To explore how poor care quality experiences for children with intellectual disability may impact on healthcare inequity, we undertook a retrospective chart review of the population of inpatient children coded with intellectual disability admitted in 2016 for greater than 23 h to two tertiary children's hospitals to calculate the differences in median length of stay for children with and without intellectual disability. This study found 336 children of the total 14 244 admitted children had at least one admission coded with an intellectual disability, according to the ICD‐10 (World Health Organisation, 2004)—Australian Modification. The intellectual disability cohort had a median length of stay of 87 h (IQR: 47–187) compared to the rest of the admitted population median of 64 h (IQR: 37–122), a significant difference (*P* value: <.001) (Mimmo et al., 2019a). Even with this underestimate in identifying children with intellectual disability, the 23‐h difference in length of stay suggests an increased level of complexity and risk for this already disadvantaged population. However, retrospective metrics such as length of stay and clinical outcomes offer a limited view of the experience of care quality. Are these measures ideal for assessing care quality and unwarranted clinical variation, or are there other underexplored avenues such as real‐time patient experience indicators that can enhance understanding of the complexities of variance and even pre‐empt unwarranted clinical variation? Using quantitative metrics alone presents a missed opportunity to understand and utilize patient experience narratives to discern, learn from and disseminate what Dadich and colleagues refer to as brilliance in care quality (Dadich et al., 2015).

Healthcare records can only tell us part of the story; much of the rich knowledge of the patient experience comes from children with intellectual disability and families. Our two reviews of literature involving children with intellectual disability and/or their parents/carers have reported poor experiences of care related to healthcare staff assumptions of the child's abilities, reliance on parents/carers to attend to the child's needs, lack of awareness of the child with intellectual disability and their care needs, and lack of effective partnerships in care (Mimmo, Harrison, & Hinchcliff, 2018; Mimmo, Woolfenden, Travaglia, & Harrison, 2019b). Parents/carers of children with intellectual disability consistently report good experiences of care with healthcare staff who know or take the time to get to know their child and their needs (Mimmo et al., 2019b).

Other hospital indicators of care quality such as 30‐day readmission, Did Not Attend appointments and Discharge Against Medical Advice rates, sepsis and inpatient falls, while can be captured through hospital incident reporting systems, do not routinely report how these measures differ for people with intellectual disability, and there is little published research in this area. Available published evidence shows this population has higher rates of avoidable and unplanned readmissions (Kelly et al., 2015). A 2019 review of patient safety for people with intellectual disabilities showed significant issues in five categories. These include (1) health conditions which are unrecognized, misdiagnosed or delayed in diagnosis; (2) finding it more difficult to utilize healthcare services; (3) having a higher likelihood of experiencing medication related safety incidents including errors in the prescription, dispensing and administration of medication, and in polypharmacy; (4) having a higher probability of experiencing complications during their care pathway and in the period that they spend in hospital compared to the general population; and (5) being more likely to experience inadequate basic health care (Travaglia et al., 2019). While inpatient mortality is another common measure of care quality, hospital mortality data have been shown to be unreliable in identifying children with intellectual disability (Bourke et al., 2018).

The development of valid patient experience measures of care quality appropriate for paediatric populations, such as Patient Reported Experience Measures, or PREMs, is progressing, and there is a need to develop PREMs that children with intellectual disability can use. However, despite the high healthcare utilization rates in the intellectual disability population, these PREMs are not applicable to children and young people (CYP) with intellectual disability:

‘Furthermore, the CYP PREMs are not suitable for CYP with moderate or severe learning disability, and further work is required to develop a PREM that is appropriate for their use in terms of both engaging them and eliciting their views.’ (Wray, Hobden, Knibbs, & Oldham, 2018, p. 104).

This is a missed opportunity to address poor experiences of care for these children and we recommend any future paediatric specific metric, such as PREMs, are developed in partnership with children with intellectual disability to increase the applicability of this metric across patient populations.

## 4 CONSUMER ENGAGEMENT AND THE VOICE OF THE CHILD WITH INTELLECTUAL DISABILITY

Children with intellectual disability have higher rates of healthcare utilization than their peers (Bebbington et al., 2013; Heslop et al., 2014) and have much to contribute on their experiences of healthcare. By excluding the voice of the child with intellectual disability from patient experience data, healthcare services overlook a swathe of rich data that comes from the experiences of these children about what matters in healthcare. In addition, listening to and acting on the healthcare experiences of children with intellectual disability can address the potential long‐term health, social and educational inequities for this group. Our previous work found healthcare staff assumptions about the behaviour and cognitive capacity of children with intellectual disability and reliance on their parents to attend to their care needs contributed to poor experiences in care quality and clinical outcomes (Mimmo et al., 2018).

**FIGURE 1:**
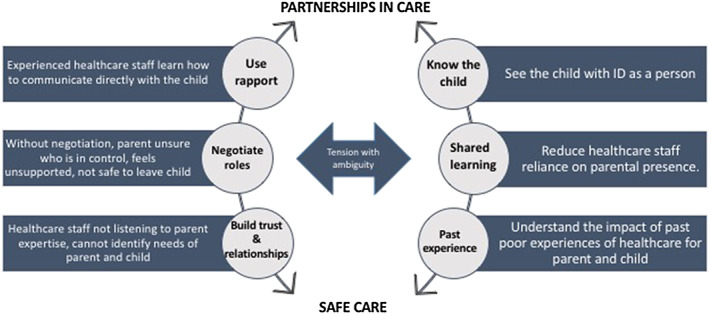
Conceptual model for safe care of a child with intellectual disability in hospital (Mimmo et al., 2019b) [Colour figure can be viewed at wileyonlinelibrary.com]

Our recently developed conceptual model of safe care for children with intellectual disability in hospital (Figure 1) (Mimmo et al., 2019b) draws upon findings from our meta‐narrative of the parental experience with a child with intellectual disability in hospital as a basis for ensuring that the voice of the consumer is central to addressing inequitable quality and safety (Mimmo et al., 2019b). The model illustrates how safe care ensues when healthcare staff develop effective partnerships with parents/carers of children with intellectual disability by negotiating roles, building trust and sharing knowledge with parents/carers to know the child with intellectual disability (Mimmo et al., 2019b) and will be tested in upcoming participatory research with children with intellectual disability in hospital.

Inclusion of children with intellectual disability to actively inform the design and development of systems of healthcare will ensure systems that can meet their needs. This will optimize equity of care quality and safety outcomes for these children. An example of this in practice is our current research to elicit the voices of children with intellectual disability regarding experiences of healthcare with co‐researchers who are young people with intellectual disability. The experiential data gathered will contribute to the co‐design of a tool for healthcare services to routinely elicit patient experience data directly from children with intellectual disability.

## 5 CONCLUSION

To enable safe, high quality, equitable care for children with intellectual disability, healthcare organizations must first apply consistent terminology and utilize their existing electronic systems to identify the children with intellectual disability who access their services, use standard care quality metrics to distinguish problematic areas for further exploration, then provide opportunity and an audience for these children to voice their experiences of healthcare and actively participate in the design and development of relevant patient experience measures that are suitable for all children. The next crucial step is to create effective partnerships with children with intellectual disability and their parents/carers and health staff. Once we identify children with intellectual disability, we need to create effective partnerships with children with intellectual disability and their parents/carers to address the inequities in care quality experiences and create safer healthcare for all children.

## RESEARCH ETHICS APPROVAL

The Sydney Children's Hospitals Network Human Research Ethics Committee, ethics no.: LNR17SCHN277.

## FUNDING INFORMATION

This work was supported by Maridulu Budyari Gumal, The Sydney Partnership for Health, Education, Research & Enterprise (SPHERE) Translational Research Fellowship Scheme [2019–2021] awarded to LM.
